# Combining Hexanoic Acid Plant Priming with *Bacillus thuringiensis* Insecticidal Activity against Colorado Potato Beetle

**DOI:** 10.3390/ijms140612138

**Published:** 2013-06-06

**Authors:** Inmaculada García-Robles, Camila Ochoa-Campuzano, Emma Fernández-Crespo, Gemma Camañes, Amparo C. Martínez-Ramírez, Carmen González-Bosch, Pilar García-Agustín, Carolina Rausell, María Dolores Real

**Affiliations:** 1 Department of Genetics, University of Valencia, Dr. Moliner 50, Burjassot 46100, Valencia, Spain; E-Mails: garciai@uv.es (I.G.-R.); camila.ochoa@uv.es (C.O.-C.); amparo.martinez@uv.es (A.C.M.-R.); carolina.rausell@uv.es (C.R.); 2 Biochemistry and Biotechnology Laboratory, Plant Physiology Area, Department CAMN, University Jaume I, Castellón 12071, Spain; E-Mails: ecrespo@guest.uji.es (E.F.-C.); camanes@uji.es (G.C.); garciap@uji.es (P.G.-A.); 3 Department of Biochemistry and Molecular Biology, University of Valencia, IATA (CSIC), Paterna, Valencia 46980, Spain; E-Mail: carmen.gonzalez@uv.es

**Keywords:** Colorado potato beetle, *Bacillus thuringiensis*, Cry3Aa toxin, intestain proteases, proteolysis, Solanaceae, hexanoic acid, priming, plant defense, plant hormones

## Abstract

Interaction between insect herbivores and host plants can be modulated by endogenous and exogenous compounds present in the source of food and might be successfully exploited in Colorado potato beetle (CPB) pest management. Feeding tests with CPB larvae reared on three solanaceous plants (potato, eggplant and tomato) resulted in variable larval growth rates and differential susceptibility to *Bacillus thuringiensis* Cry3Aa toxin as a function of the host plant. An inverse correlation with toxicity was observed in Cry3Aa proteolytic patterns generated by CPB midgut brush-border membrane vesicles (BBMV) from Solanaceae-fed larvae, being the toxin most extensively proteolyzed on potato, followed by eggplant and tomato. We found that CPB cysteine proteases intestains may interact with Cry3Aa toxin and, in CPB BBMV from larvae fed all three Solanaceae, the toxin was able to compete for the hydrolysis of a papain substrate. In response to treatment with the JA-dependent plant inducer Hexanoic acid (Hx), we showed that eggplant reduced OPDA basal levels and both, potato and eggplant induced JA-Ile. CPB larvae feeding on Hx-induced plants exhibited enhanced Cry3Aa toxicity, which correlated with altered papain activity. Results indicated host-mediated effects on *B. thuringiensis* efficacy against CPB that can be enhanced in combination with Hx plant induction.

## 1. Introduction

The Colorado potato beetle (*Leptinotarsa decemlineata* Say, family Chrysomelidae, CPB) has proven to be an extremely difficult pest to manage because of its ability to develop insecticide resistance. Thus, integrating multiple control techniques appears to be the only sustainable way to protect susceptible crops from this exceptionally destructive insect pest for potato (*Solanum tuberosum*, Solanaceae), tomato (*Solanum lycopersicum*, Solanaceae), eggplant (*Solanum melongena*, Solanaceae), pepper (*Capsicum annuum*, Solanaceae), and other solanaceous crops in Europe, and, North and Central America [1,2].

Combining different, but potentially complementary toxicity mechanisms has been shown to provide effective plant disease control [3]. An environmentally sound tactic for the integrated pest management of important agronomic insect pests might be the use of host-plant resistance and microbial insecticides. *Bacillus thuringiensis* (family Bacillaceae, Bt) represents the most important viable microbial agent for the biological control of CPB. The insecticidal activity of Bt relies on insecticidal proteins secreted during vegetative growth (vegetative insecticidal proteins, called Vip proteins) and on parasporal crystalline protoxins (Cry and Cyt proteins). Cry toxins delivered as conventional sprayable bioinsecticides or engineered in transgenic plants have been successfully used as bioinsecticides for decades. To exert their toxic effect, after ingestion by susceptible insects, Cry proteins are proteolytically activated by gut proteases and the active toxin binds to receptors located on the brush border midgut epithelium. Breakdown of the gut epithelium through a pore formation mechanism on the target membrane or an alternative cell death process involving the adenylyl cyclase/PKA signalling pathway have been proposed as responsible for Cry cytotoxic effect [4,5].

The potential exploitation of plant endogenous resistance mechanisms for crop protection as a complement to the use of Bt toxins for biological control might offer a good strategy for the integrated management of insect pests, as was the case of the naturally occurring defensive plant cysteine protease Mir1-CP that synergizes the effects of Bt toxin Cry2A and provides an effective pest control [6]. Stacking resistance genes with Bt should also reduce the likelihood for development of insect resistance to this type of toxin and result in more sustainable pest control.

It has been documented that the general response of the host plant to specific stress stimuli resulting in the accumulation of distinct sets of defense-related compounds in leaf tissues also induce important alterations in insect physiology [7], which in turn can impact insect susceptibility to insecticides such as Bt based bioinsecticides [8]. Plant resistance is regulated by a complex network of signal molecules and transcriptional regulators, in which the plant hormones salicylic acid (SA), jasmonic acid (JA), ethylene (ET) and abscisic acid (ABA) play a crucial role [9]. Hexanoic acid (Hx) is a natural compound, inducer of plant defenses by means of a jasmonic acid (JA)-dependent priming mechanism that acts differentially, depending on the pathogen. Hx protects *S. lycopersicum* and *Arabidopsis thaliana* (Brassicaceae) plants against the necrotroph *Botrytis cinerea* (Sclerotiniaceae) [10,11], significantly inducing oxylipin 12-oxo-phytodienoic acid (OPDA) and the bioactive molecule jasmonoyl-l-isoleucine JA-Ile, and enhancing callose accumulation. In contrast, Hx-induced resistance (Hx-IR) against the hemibiotrophic pathogen *Pseudomonas syringae* (Pseudomonadaceae) alters the oxylipin pathway by reducing the accumulation of the active molecule JA-Ile and by increasing OPDA accumulation, and also primes SA signaling [12]. Very recently, the effectiveness of Hx in the control of *Alternaria alternata* (Pleosporaceae) via the JA signaling pathway in Fortune mandarin was also reported [13].

Insect herbivores activate induced plant defenses via the same signaling pathways used by plants to protect themselves from microbial pathogens, with the jasmonate family of lipid-derived signals playing a prominent role in promoting plant resistance to herbivores. JA is one of the best studied phytohormones, involved in responses to various stresses, such as attack from herbivores and necrotrophic fungi [14–17]. JA can either be methylated into methyl jasmonate (MeJA) by JA carboxylmethyltransferase, or conjugated with amino acids, particularly Ile (JA-Ile), catalyzed by JAR enzymes [18,19]. Importantly, JA-Ile has been identified as the molecule that activates the majority of JA-induced responses [20,21].

It has been demonstrated that CPB larvae fed plants treated with MeJA partially compensate for defense-related proteins by adapting their digestive proteolytic system [7]. Moreover, it has been reported that the nature of the digestive proteolytic system in CPB larvae is in part determined by the type of diet consumed [22]. On this basis, the objective of this study was to assess the effect of the plant priming inducer Hx on the Bt insecticidal activity against CPB larvae fed on three solanaceous plants (potato, eggplant and tomato).

## 2. Results and Discussion

### 2.1. Host Plant Modifies the Growth of CPB Larvae, Affecting their Susceptibility to Bt Cry3Aa Toxin

Interaction between insect herbivores and host plants can be modulated by an enormous variety of both endogenous and exogenous compounds present in the source of food.

Previous studies carried out on CPB larvae demonstrated that, depending on the rearing diet, larvae exhibited variable growth rates, similar for potato and eggplant diets but lower for the tomato diet [22]. We performed feeding tests with these three solanaceous host plants and, while CPB larvae reared on potato plants had a significantly higher weight than larvae reared on either eggplant or tomato plants, no statistically significant differences in weight between larvae fed on either eggplant or tomato plants were found (Table 1). Potato-fed CPB larvae of an initial weight of 50 mg/individual, at day 7 weighed 54% and 64% more than those fed on eggplant or tomato, respectively (Table 1). Therefore, in our experimental conditions, relative to eggplant and tomato plants, potato plants provided the best food resource for CPB growth.

Additionally, we assessed Cry3Aa toxicity in CBP larvae and statistically significant differences in Cry3Aa toxin mortality among CPB larvae reared on different solanaceous plants were observed when challenged with this toxin (Table 1), indicating that host plant influences toxin susceptibility. Treatment with a 4.3 pmol Cry3Aa toxin dose caused the highest mortality in tomato-fed larvae (89%) showing that proteins and secondary metabolites present in tomato increase Bt efficacy in relation to potato or eggplant-fed larvae. Therefore, the host plant modifies the interaction between Bt and the target insect, as previously reported in *Malacosoma disstria* (Lasiocampidae) and *Trichoplusia ni* (Noctuidae) [23,24].

### 2.2. Effect of CPB Larvae Diet on Cry3Aa Toxin Proteolysis

**Figure 1:**
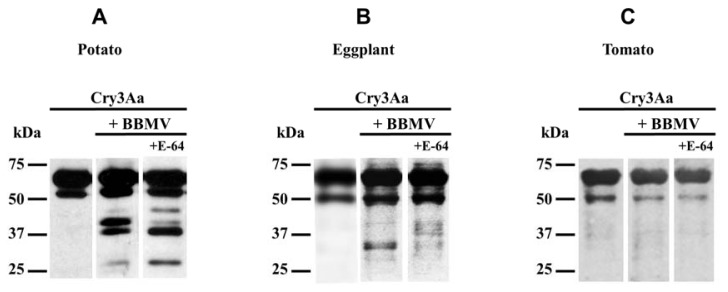
Polypeptide profile of Cry3Aa toxin cleaved by CPB BBMV midgut proteases. All panels show Western blots of Cry3Aa toxin incubated with CPB BBMV in the absence or presence of the cysteine protease inhibitor E-64, and immunodetected with an anti-Cry3Aa toxin polyclonal antibody. (**A**) BBMV from potato-fed CPB larvae; (**B**) BBMV from eggplant-fed CPB larvae; (**C**) BBMV from tomato-fed CPB larvae. In each panel, molecular weight markers are shown on the left of each blot.

The diet-related plasticity of the digestive proteolytic system in CPB larvae is well known [22]. Since toxin activation by insect midgut proteases is the first critical step in Bt toxin’s mode of action [4], we analyzed the Cry3Aa toxin proteolytic patterns generated by midgut proteases from CPB larvae fed on different host plants to seek differences which might account for changes in Cry3Aa toxin efficacy (Figure 1).

In CPB larvae, protein digestion is mainly accomplished by cysteine proteases combined with aspartic proteases, although other minor proteolytic activities such as serine proteases (chymotrypsin-like) and metalloproteases (carboxypeptidase A-like and leucine aminopeptidase-like) have been also detected in the midgut of CPB larvae [25,26]. Therefore, the effect of the cysteine protease inhibitor E-64 on Cry3Aa toxin proteolysis by CPB BBMV was also assessed.

Consistent with the reported influence of diet on the accumulation of specific proteinase forms in the CPB midgut [22], the Cry3Aa toxin proteolytic pattern varied considerably among the three host plants studied. An inverse correlation with toxicity was observed, being the toxin most extensively proteolyzed on potato, followed by eggplant and tomato. Moreover, the effect of the E-64 inhibitor on Cry3Aa toxin proteolysis by BBMV from potato or eggplant-fed CPB larvae (Figure 1) corroborates that cysteine proteases might play a role in this toxin mode of action. There is good evidence that changes in midgut protease activity correlate with Cry toxin resistance, not only in CPB [27], but also in other insect pests, such as the Indianmeal moth *Plodia interpunctella* (Pyralidae) [28], the tobacco budworm *Heliothis virescens* (Noctuidae) [29], the cotton bollworm *Helicoverpa armigera* (Noctuidae) [30], and the mosquito *Aedes aegypti* (Culicidae) [31]. In the western corn rootworm *Diabrotica virgifera virgifera* (Chrysomelidae), an engineered chymotrypsin/cathepsin G site in domain I rendered Bt Cry3Aa toxin active against these insect larvae [32].

Lower Cry3Aa toxin cleavage by BBMV from eggplant and tomato-fed CPB larvae prompted us to speculate that the reduced activity of gut proteases on Cry3Aa toxin might result in increased susceptibility due to a higher availability of non-degraded toxin. Altered toxin proteolysis has been reported to lead to decreased Cry toxicity, as in a Cry1Ac toxin resistant *H. armigera* strain, in which resistance to Bt Cry1Ac toxin was due to improper processing of the protoxin [30]. Therefore, knowledge of the potential role of proteases on Cry toxins might be relevant to elucidate the mechanisms involved in the enhancement of Bt toxicity and offer an important tool in resistance management.

### 2.3. Identification of Cry3Aa Interacting Cysteine Proteases

**Figure 2:**
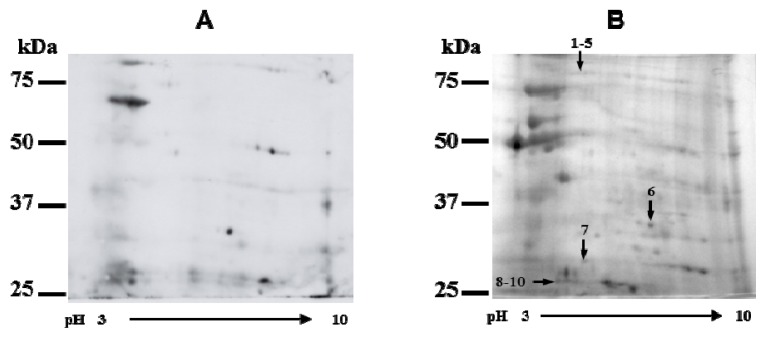
Identification of CPB midgut cysteine proteases that interact with Cry3Aa toxin. (**A**) Bt Cry3Aa toxin biotin-labeled with the trifunctional crosslinking reagent Sulfo-SBED was incubated with CPB BBMV, and following photoactivation, DTT was added. The biotin label was transferred from Cry3Aa toxin to CPB BBMV binding proteins and detected by 2D-Western blot; (**B**) Coomassie stained CPB BBMV proteins separated by 2D-electrophoresis. Arrows 1 to 10 indicate the bands excised from the Coomassie stained gel that gave a positive match following trypsin digestion and mass spectroscopy analysis. Molecular weight markers are shown on the left in each panel.

To identify CPB midgut cysteine proteases that may interact with Cry3Aa toxin, a photoactive cross-linking approach with sulfo-SBED was implemented using BBMV from potato-fed larvae that readily proteolyzed Cry3Aa toxin. In this type of design the toxin was first derivatized with sulfo-SBED and allowed to interact with proteins from CPB midgut BBMV. Following UV-induced crosslinking between interacting proteins, and subsequent reduction and cleavage by DTT treatment, the biotin tag was transferred to the Cry3Aa interacting proteins in CPB BBMV. Biotin-labeled putative Cry3Aa toxin binding proteins were identified by 2-D electrophoresis and Western blotting analysis using horseradish peroxidase-streptavidin (Figure 2). Spots were excised from the corresponding gel, trypsin digested, and peptide fragments analyzed by mass-spectrometry (MALDI TOF/TOF, or LC MS/MS when necessary) to obtain the peptide mass fingerprinting (PMF). Since the CPB genome is not sequenced, identifications were performed by sequence similarity searching the non-redundant protein database at NCBI (taxonomic restriction to insects) with PMF data using Mascot (MatrixScience, Ltd., London, UK) to establish the best protein match for the spots. Despite obtaining high quality mass spectra for many of the proteins, a considerable number of them were not identified, most probably due to the lack of CPB sequences in NCBI database. A Mascot score lower than 80 was considered a non-significant hit, except for spot 1 in which the significance threshold was 52. In Figure 2B, arrows point to spots that gave a positive match and Table 2 shows the corresponding identifications, in which spot 9 proteins with the best matches, although slightly below the threshold score, were also included. Among the identified proteins, spots 8 to 10 corresponded to CPB intestains, major digestive papain type cysteine proteases in CPB [33].

**Figure 3:**
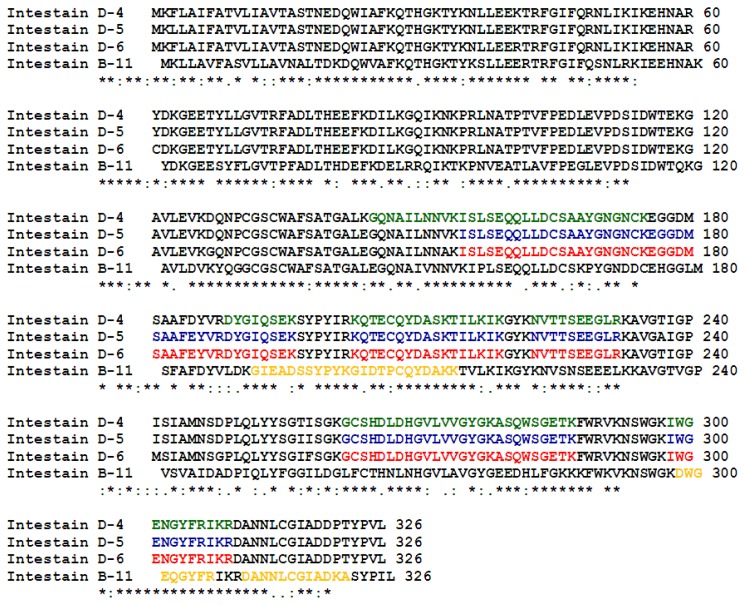
Multiple sequence alignment of CPB intestains D-4, D-5, D-6 y B-11 using Clustal omega [36]. Colored amino acids depict mass spectrometry peptides identified for each protein.

Among the six subfamilies included in the proposed classification for the intestain family [34] we only found instestains belonging to IntB and IntD subfamilies. The amino acid identity is more than 91% within IntB subfamily [35] and members of the IntD subfamily have 85%–95% of their amino acids in common [26]. Both subfamilies are 61%–76% identical at the amino acid level and 30%–45% identical to different mammalian cathepsins [26,35]. The highly amino acid conservation among the CPB intestains identified as putative Cry3Aa toxin interacting proteases whose complete coding sequence is available in NCBI database can be observed in the multiple sequence alignment shown in Figure 3, in which the mass spectrometry peptides found for each protein are indicated.

Mass spectrometry identification of CPB intestains among BBMV Cry3Aa toxin interacting proteins supports the involvement of this type of cysteine protease in the proteolytical cleavage of Cry3Aa toxin.

### 2.4. Analysis of Papain Type Cysteine Protease Activity in CPB Larvae Fed on Different Host Plants

**Figure 4:**
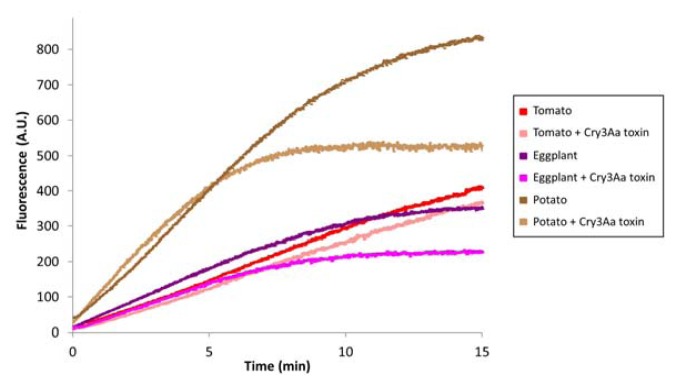
Analysis of papain type cysteine protease activity in BBMV from potato, eggplant and tomato-fed larvae in the absence or presence of Cry3Aa toxin. Points are the mean of two replicates (*n* = 2).

To further confirm the role of the papain type of cysteine protease intestains in Cry3Aa toxin mode of action in CPB, papain activity was monitored in CPB BBMV from potato, eggplant, and tomato-fed larvae using the Z-Phe-Arg-MCA substrate, and assays were performed in the presence or absence of Cry3Aa toxin as competitor of substrate hydrolysis (Figure 4). Independently of the solanaceous plant ingested by the insect, we detected papain activity associated with CPB BBMV and Cry3Aa toxin was able to compete for the hydrolysis of the substrate. The highest activity was found in BBMV from potato-fed larvae, in which 40% of papain activity was competed by Cry3Aa toxin. Papain activity was similar in BBMV from eggplant and tomato-fed larvae (around 50% of papain activity in BBMV from potato-fed larvae). Nevertheless Cry3Aa toxin competition for substrate hydrolysis was significantly higher in BBMV from eggplant than in tomato-fed larvae. These results are in accordance with Cry3Aa toxin being more proteolyzed by BBMV from potato-fed CPB larvae followed by BBMV from eggplant-fed larvae and to a much lesser extent by BBMV from tomato-fed larvae (Figure 1).

### 2.5. Effect of Plant Hx Treatment on Cry3Aa Toxin Insecticidal Action against CPB Larvae

It has been previously demonstrated that CPB larvae differentially adapt their digestive protease complement to the presence of distinct sets of defense compounds in potato leaves induced with the chemical effector MeJA [7].

CPB intestains B and D detected as Cry3Aa toxin interacting proteases were first identified in CPB midgut extracts of larvae grown on MeJA-induced potato leaves [26,33], in which cysteine proteinase activities insensitive to potato proteinase inhibitors were reported to be induced [37], suggesting that intestains are key enzymes in the mechanism of insect adaptation to the plant defense system. We hypothesized that if intestains were involved in Cry3Aa toxin action, changes in the composition of digestive proteases in CPB larvae fed on induced plants might influence Cry3Aa toxicity. Therefore, we undertook a study to determine if the plant defense JA-dependent inducer Hx affects Cry3Aa toxin efficacy against CPB in relation to the data presented in sections 2.1 to 2.4, in which larvae were fed with non Hx-induced plants.

#### 2.5.1. Cry3Aa Mortality of CPB Larvae Fed on Three Hx-induced Solanaceous Plants

**Figure 5:**
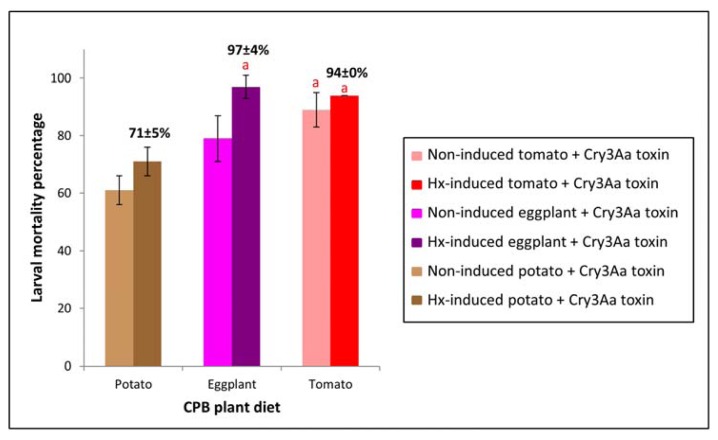
Mortality percentage of CPB larvae fed Hx-induced potato, eggplant or tomato versus CPB larvae fed non-induced plants, following challenge with Cry3Aa toxin. Error bars represent standard error of the mean of three biological replicates (*n* = 3) of each sixteen CPB larvae. Means followed by the same letter were not significantly different (Student’s *t*-test, *p* > 0.05).

Statistically significant higher Cry3Aa toxin mortality was observed in CPB larvae reared on Hx-induced eggplant and potato plants compared to CPB larvae fed non-induced eggplant and potato plants (Figure 5), demonstrating that Hx host plant treatment is modifying CPB larvae susceptibility to Cry3Aa toxin. In Hx-induced tomato-fed larvae, although increased mortality was also observed relative to larvae fed non-induced tomato plants (Figure 5), results were not statistically significant, probably due to the fact that percentage mortality of CPB larvae feeding on non-induced tomato was already close to 90% after toxin treatment.

Combining induced resistance and other biological approaches has been reported to be successful in the control of diverse pathogens. On tomato, the chemical inducer acibenzolar-*S*-methyl (ASM) used in combination with *Pseudomonas fluorescens* (Pseudomonadaceae) Pf2 or four plant growth promoting rhizobacteria strains of *Bacillus* sp., provided more efficient control of *Ralstonia solanacearum* (Ralstoniaceae) or Fusarium crown and root rot, respectively [38,39].

#### 2.5.2. Feeding Performance of CPB Larvae Fed on Three Hx-Induced Solanaceous Plants

Induction of host plant resistance may make insect pests more susceptible to insecticides by retarding insect growth and development [40], as it was the case of *Spodoptera littoralis* (Noctuidae) [41].

**Figure 6:**
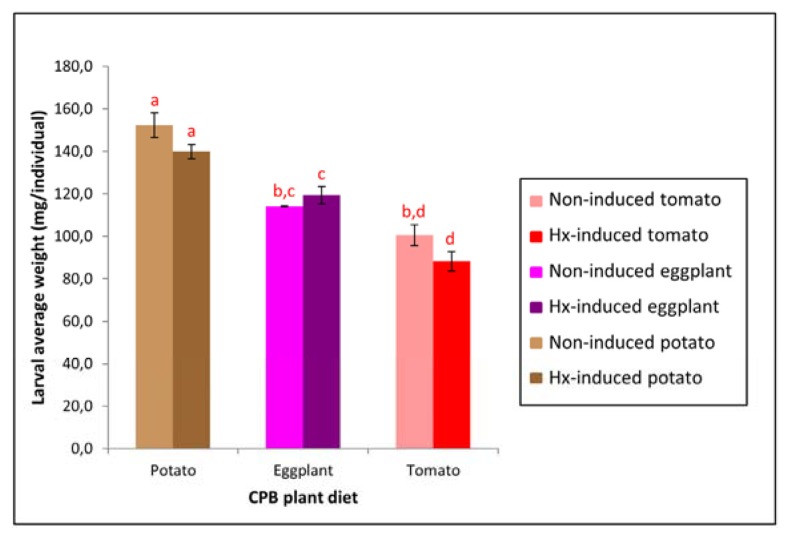
Mean larval weight of CPB larvae fed Hx-induced potato, eggplant or tomato versus CPB larvae fed non-induced plants. Error bars represent standard error of the mean of three biological replicates (*n* = 3) of each sixteen CPB larvae. Means followed by the same letter were not significantly different (Student’s *t*-test, *p* > 0.05).

When we reared CPB larvae on potato, eggplant and tomato Hx-induced plants, although larval weight differences in relation to insects fed on the corresponding non-induced plants were not statistically significant, larval weight of larvae reared on induced potato and tomato plants was slightly lower compared to those of insects reared on non-induced plants, whereas larval weight of larvae reared on induced eggplants was slightly higher than that of insects reared on non-induced eggplants (Figure 6). Therefore, increased susceptibility to Cry3Aa toxin in larvae fed Hx-induced plants (Figure 5) was not due to larvae being less well-nourished than those fed on non-induced plants and factors other than retarded insect growth are making CPB larvae less tolerant to Cry3Aa toxin.

#### 2.5.3. Cysteine Protease Activities on CPB Larvae fed on three Hx-induced Solanaceous Plants

**Figure 7:**
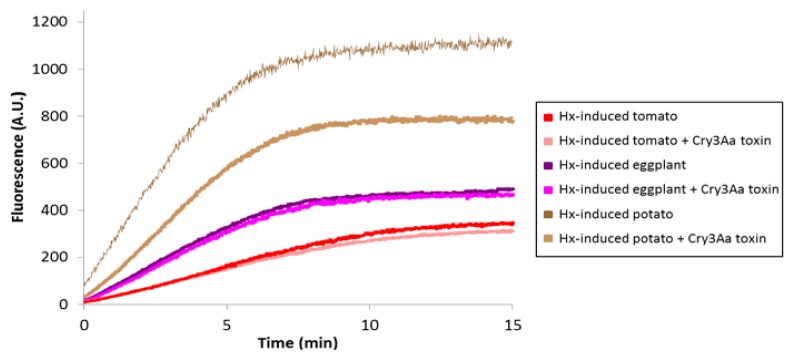
Analysis of papain type cysteine protease activity in BBMV from Hx-induced potato, eggplant and tomato-fed larvae in the absence or presence of Cry3Aa toxin. Points are the mean of two replicates (*n* = 2).

Increased Cry3Aa toxin susceptibility in larvae fed Hx-treated plants might be related to variations in the digestive protease complement. Therefore, papain type cysteine protease activities were monitored in CPB BBMV from Hx-induced potato, eggplant, and tomato-fed larvae using Z-Phe-Arg-MCA as a substrate, and the ability of Cry3Aa toxin to compete for the substrate hydrolysis was analyzed (Figure 7).

Remarkably, when comparing with papain activity in CPB BBMV from non-induced plant fed-larvae (Figure 4), in BBMV from Hx-induced eggplant and tomato-fed larvae, Cry3Aa toxin lost the ability to compete for the substrate hydrolysis. In BBMV from Hx-induced potato-fed larvae, although papain activity was significantly higher than in BBMV from non-induced potato-fed larvae, Cry3Aa toxin competition for the substrate hydrolysis was slightly reduced to 30%, and variation in the shape of the curves was indicative of a change in substrate specificity in response to the quantity and type of compounds induced in potato by Hx (compare Figures 4 and 7). These observations are consistent with Cry3Aa toxin decreased degradation being in part responsible for increased larval susceptibility to the toxin in all host plants. Results suggest that CPB adaptation to changes in Hx-induced plants could follow a common pattern for the three Solanaceae.

### 2.6. Study of the Hormonal Pattern in Eggplant and Potato Plants Following Hx Induction

**Figure 8:**
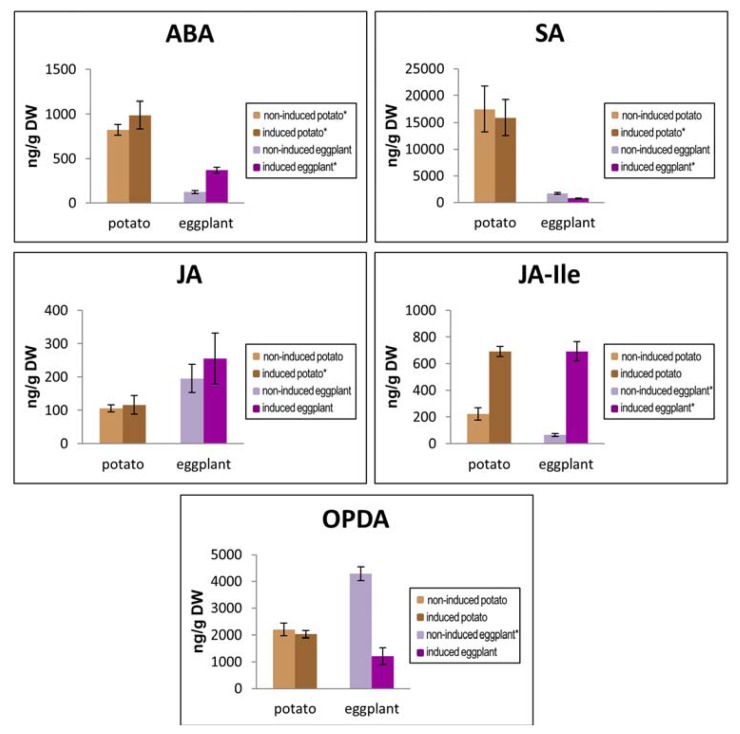
Hormone levels in non-induced and Hx-induced eggplant and potato plants. Leaves were collected from non-induced plants and 48 h after plant Hx induction. Abscisic acid (ABA), salicylic acid (SA), jasmonic acid (JA), jasmonoyl-l-isoleucine (JA-Ile) and 12-oxo-phytodienoic acid (OPDA) levels were determined in lyophilized material by high performance liquid chromatography mass spectrometry. Data are the mean ± standard error of two (*n* = 2), or three experiments (*n* = 3) in determinations labeled with an asterisk in the color key of graph legends.

Host genotype is known to affect the expression of induced resistance [3]. Therefore, we examined the influence of the type of solanaceaous CPB host plant on the hormonal pattern induced by Hx treatment. Since we have previously reported that JA-signaling mechanism is involved in Hx-IR in tomato plants [10], in this work, we analyzed the hormonal levels in non-induced and Hx-treated eggplant and potato plants (Figure 8) to investigate the mechanism underlying the Hx host plant effect.

The basal hormone levels of ABA, SA, JA-Ile and OPDA differed significantly between eggplant and potato plants, with potato plant ABA, SA and JA-Ile, 7-fold, 10-fold and 2-fold, respectively, those of eggplant. However, the basal OPDA level in eggplant was 2-fold that of potato plants, and JA basal levels were not statistically significantly different between both plants.

The hormonal profile induced by Hx showed a common pattern in the two solanaceous plants, increasing ABA, JA and JA-Ile, and decreasing SA and OPDA (Figure 8). Nevertheless, the observed differences in relation to non-induced plants were not statistically significant in all cases. Hx treatment of potato plants only significantly induced JA-Ile levels, whereas in eggplant plants, induction significantly altered all the hormones assayed, except JA. Interestingly, JA-Ile levels were significantly increased upon Hx-induction, both in eggplant and potato plants, whereas a remarkable statistically significant decrease of OPDA in Hx-induced eggplants was observed, reinforcing the relevance of oxylipin signaling pathway in Hx-IR in these Solanaceae.

Priming agents and wounding can be used to boost plant resistance to herbivores [41] and some examples for primed responses can be found in the literature, all requiring that the plants be previously challenged by either nonpathogenic strains of potential pathogens or treated with chemicals or analogs of those chemicals that are usually involved in defense-response signaling. The main advantage of this kind of defense sensitization is that provides a cost-efficient strategy that does not affect the general physiology of the plant, as the full induction of all possible plant countermeasures would do [42].

Hx priming of defense has been effective in the context of plant-pathogen interaction against a broad spectrum of plant attackers [10–13]. In this work, we demonstrated that Hx priming induced changes that can modulate plant resistance to herbivores. Strategies for protecting crops from insect pests, based on the exploitation of endogenous resistance mechanisms exhibited by plants to insect herbivores, are an exciting possibility for biological control of pest species but it has shown only a marginal effectiveness [40]. Combining host plant resistance factors with other pest control strategies can increase plant protection.

Overall, our data corroborate the diet-related plastic response of CPB larvae that adapt their digestive proteases to the type of host plant ingested and also to the presence of compounds and specific defense molecules induced by the natural priming agent Hx. In response to Hx treatment, CPB larvae feeding on induced Solanaceae plants adapt to the new composition of the feeding source by means of specific digestive proteases that in turn influence larval susceptibility to Bt toxins.

Although induction of gut proteases that are not sensitive to the plant inducer may represent a commonly occurring adaptive mechanism among insects, specifically reported in CPB [36], the Hx effect is particularly interesting. CPB larvae fed on Hx-induced plants were not able to overcome the effects of the compounds induced by Hx and, upon Cry3Aa toxin treatment, increased larval mortality was observed compared to CPB larvae fed on non-induced plants challenged with the toxin.

Novel insight into host-mediated effects on Bt efficacy might be turned into new tools to engineer durable, broad-spectrum plant protection improving CPB pest management and delaying insect resistance development.

## 3. Experimental Section

### 3.1. Plants

Potato plants (*Solanum tuberosum* L. cv. Vivaldi), eggplant plants (*Solanum melongena var. esculentum* cv. Black Round) and tomato plants (*Solanum lycopersicum* Mill cv. Ailsa Craig) were used. Plants were grown under greenhouse conditions at day and night temperatures of 22 ± 4 °C and 20 ± 4 °C, respectively, with 16 h of light and 8 h of darkness and RH = 60%.

### 3.2. Insects

A laboratory colony of *Leptinotarsa decemlineata* (Colorado potato beetle) founded from eggs taken from the field was used. Adults were reared on greenhouse grown potato plants at 25 ± 1 °C and with a photoperiod of 16:8 h (light/dark). After hatching, larvae were transferred either to potato, tomato or eggplant fresh leaves. New leaves were supplied to the larvae every 24 h.

### 3.3. Hexanoic Acid Plant Defense Induction

To induce plant defenses, pots (748 cm^3^) containing potato, eggplant or tomato plants were watered with 50 mL of 20 mM hexanoic acid. After 48 h, induced plants or fresh leaves were used for feeding insects or performing insect mortality assays.

### 3.4. Preparation of Brush Border Membrane Vesicles (BBMV)

BBMV were prepared from last instar CPB larvae according to the method of Wolfersberger *et al*. [43], as modified by Reuveni and Dunn [44]. Larvae were dissected in storage buffer (300 mM mannitol, 20 mM 2-mercaptoethanol, 5 mM EGTA, 1 mM EDTA, 10 mM HEPES, pH 7.5) and the insect midguts obtained were immediately frozen and stored at −80 °C until use. Frozen midguts were mechanically homogenized in homogenization buffer (200 mM mannitol, 10 mM ascorbic acid, 5 mM EDTA, 2 mM DTT, 10 mM HEPES, pH 7.4) for 10 s. One volume of 24 mM MgCl_2_ was added and the mixture was incubated for 10 min. Following centrifugation of the mixture (10 min, 6000× *g* at 4 °C), the supernatant was further centrifuged (30 min, 30,000× *g* at 4 °C) and the final pellet suspended in 200 mM mannitol, 1 mM DTT, 1 mM HEPES-Tris, pH 7.4, frozen and stored at −80 °C until use. The protein concentration of BBMV was measured by Bradford’s procedure [45] using bovine serum albumin (BSA) as a standard.

### 3.5. Toxin Purification

Cry3Aa toxin was produced in Bt strain BTS1. Crystal inclusions were separated from spores and cell debris by centrifugation in discontinuous 67%, 72%, 79%, and 84% (*w*/*v*) sucrose gradients in 50 mM Tris-HCl, pH 7.5, as described by Thomas and Ellar [46]. Crystal proteins were solubilized in 50 mM Na_2_CO_3_, pH 10.5, at 37 °C for 2 h. Purity of the crystal preparation was monitored by phase contrast microscopy and analyzed by 10% SDS-PAGE. Protein concentration was measured by the protein-dye method of Bradford [45], using BSA as a standard.

### 3.6. Cry3Aa Toxin Cleavage Assays

Proteolysis assays on Cry3Aa toxin were performed as described before [47]. Toxins (2 μM) were incubated with 20 μg BBMV in a final volume of 30 μL PBS (8 mM Na_2_HPO_4_, 2 mM KH_2_PO_4_, 150 mM NaCl, pH 7.4), for 10 min at room temperature, and centrifuged 20 min at 12,000× *g*. The supernatants were loaded in 10% SDS-PAGE gels and the resolved proteins were transferred onto a nitrocellulose membrane (Millipore, Billerica, MA, USA) and immunoblotted against anti-Cry3Aa polyclonal antibody. The secondary antibody was alkaline phosphatase-conjugated anti-rabbit antibody (Sigma, St. Louis, MO, USA). The immunoreactive proteins were visualized using the ECL detection system Immobilon Western (Millipore, Billerica, MA, USA).

In toxins cleavage inhibition assays, CPB BBMV were incubated with E64 (20 μM) for 5 min on ice prior to toxin addition.

### 3.7. Insect Toxicity Assay

Toxicity assays were performed on newly molted third instar larvae fed non-induced or Hx-induced plants, and starved for 2 h prior to toxin treatment. Sixteen larvae were force fed with 0.3 μL of 14.4 μM Cry3Aa toxin (corresponding to 4.3 pmol Cry3Aa toxin dose) and, upon ingestion, larvae were transferred to plants either watered or not with hexanoic acid. Larval weight was recorded at the beginning of the experiments and 7 days after the initial treatment. Assays were performed in triplicate and in each case a control with larvae fed without toxin was included.

### 3.8. Determination of ABA, SA, OPDA, JA and JA-Ile Levels

Fresh plant material either from non-induced plants or 48 h after induction was frozen in liquid nitrogen and lyophilized. Before extraction, a mixture of internal standards containing 100 ng [^2^H_6_]-ABA, 100 ng [^2^H_4_]-SA and 100 ng of dihydrojasmonic [48] was added. Dry tissue (0.05 g) was immediately homogenized in 2.5 mL of ultrapure water. After centrifugation (5000× *g*, 40 min), the supernatant was recovered and adjusted to pH 2.8 with 6% acetic acid, and subsequently partitioned twice against an equal volume of diethyl ether. The aqueous phase was discarded, and the organic fraction was evaporated in a Speed Vacuum Concentrator (Eppendorf, Hamburg, Germany) at room temperature and the solid residue re-suspended in 1 mL of a water/methanol (90:10) solution and filtered through a 0.22 μm cellulose acetate filter. A 20 μL aliquot of this solution was then directly injected into the HPLC system. Analyses were carried out using a Waters Alliance 2690 HPLC system (Milford, MA, USA) with nucleosil ODS reversed-phase column (100 × 2 mm i.d.; 5 mL; Scharlab, Barcelona, Spain). The chromatographic system was interfaced to a Quatro LC (quadrupole-hexapolequadrupole) mass spectrometer (Micromass, (Micromass, Inc., Beverly, MA, USA). The MassLynx NT version 3.4 software (Micromass, Beverly, MA, USA) was used to process the quantitative data from calibration standards and the plant samples. The MassLynx NT software version 4.1 (Waters-Micromass, Manchester, UK) was used to process the quantitative data from calibration standards and the plant samples.

### 3.9. Photoactive Biotin Label Transfer Assays

The sulfosuccinimidyl-2-[6-(biotinamido)-2-(*p*-azidobenzamido) hexanoamido] ethyl-1,3′-dithiopropionate (Sulfo-SBED) reagent was used following manufacturer’s instructions (Pierce, Rockford, IL, USA). Cry3Aa toxin (50 μg) was labeled with Sulfo-SBED in a 1:3 molar ratio using the ProFound™ Sulfo-SBED Biotin Label Transfer kit (Pierce, Rockford, IL, USA). Five micrograms of Sulfo-SBED labeled Cry3Aa toxin were incubated with 30 μg of CPB BBMV for 1 h in the dark at room temperature. Following incubation, crosslinking reaction was achieved by UV irradiation on ice for 5 min, using a 75 W UV-lamp located at 5 cm from the mixture. After cleaving the disulfide bond by DTT addition, the Sulfo-SBED-cross-linked BBMV proteins were analyzed by SDS-10% PAGE or two-dimensional electrophoresis.

To perform two-dimensional electrophoresis, Sulfo-SBED labeled BBMV proteins were precipitated using the Plus One 2-D Clean-Up Kit (GE Healthcare, Little Chalfont, UK) and re-suspended in buffer containing 30 mM Tris–HCl, pH 8.5, 7 M urea, 2 M thiourea and 4% (*w*/*v*) CHAPS. The protein concentration was determined by the RC-DC Protein Assay (Bio-Rad, Hercules, CA, USA). The first dimension, isoelectric focusing (IEF), was performed in a 7 cm × 3 mm Immobiline™ Dry Strip (GE Healthcare) with a NL pH 3–10 gradient. The strip was rehydrated with a solution containing 7 M urea, 2 M thiourea, 2% CHAPS, 20 mM DTT, 1.2% (*v:v*) Destreak (GE Healthcare) and 0.5% Pharmalyte 3–10 NL. Following overnight rehydration, 0.5 mg of Sulfo-SBED labeled BBMV proteins were loaded onto the strip using a cup loading technique. IEF was performed at 50 μA per strip at 20 °C, in a voltage step-gradient (500 V, 0.30 h; 1000 V, 0.30 h; gradient 3000 V, 1 h; gradient 5000 V, 1 h; 5000 V, 1.30 h; 500 V, 1.00 h ), using an Ettam IPGPhor isoelectric focusing system (GE Healthcare, Little Chalfont, UK). Prior to the second dimension (SDS-PAGE), the strip was equilibrated for 15 min in a solution containing 50 mM Tris-HCl buffer, pH 8.6, 6 M urea, 30% glycerol, 2% SDS, 1% DTT, followed by 15 min incubation with 50 mM Tris-HCl buffer, pH 8.6, 6 M urea, 30% glycerol, 2% SDS and 2.5% iodoacetamide. After equilibration the strip was applied to 10% (*w*/*v*) polyacrylamide-SDS.

Following SDS-10% PAGE or two-dimensional electrophoresis, Sulfo-SBED labeled BBMV proteins were electrotransferred to nitrocellulose (GE Healthcare) membranes using a Bio-Rad blotting unit. Membranes were blocked for 30 min at room temperature with 3% (*w*/*v*) BSA in PBS buffer (8 mM Na_2_HPO_4_, 2 mM KH_2_PO_4_, 150 mM NaCl, pH 7.4) containing 0.1% (*v*/*v*) Tween 20. The blocked membranes were incubated for 1 h, at room temperature, with 20 ng/mL streptavidin coupled to horseradish peroxidase in PBS buffer, pH 7.4. The biotinylated proteins were visualized using a chemiluminescent reagent (Pierce, Rockford, IL, USA).

Protein spots of interest were excised from the corresponding Coomassie stained gels and trypsin digested. Peptide fragments were analyzed by mass-spectrometry (MALDI TOF/TOF) or LC MS/MS when necessary.

### 3.10. Papain Type Cysteine Protease Assays in CPB Larvae fed on Different Host Plants

BBMV papain proteolytic activity was monitored using a fluorescence cleavage assay with the synthetic peptide Z-Phe-Arg-MCA (Sigma, St. Louis, MO, USA) as a substrate. Assays were carried out with 150 μg BBMV in 1 mL of 50 mM Tris-HCl buffer, pH 7, with 10 mM freshly prepared l-cysteine as an activator, and 1 μL of substrate (20 mM in methanol) at 25 °C into the assay cuvette. Cry3Aa toxin was used as competitor in the fluorogenic substrate cleavage assay by adding 75 μg of toxin previously to the addition of the substrate. Hydrolysis rates were monitored for 20 min at λ excitation 360 nm and λ emission 450 nm, using a VARIAN Cary Eclipse Fluorescence Spectrophotometer equipped with a constant-temperature water bath.

## 4. Conclusions

Results corroborate the diet-related plasticity of CPB larvae adapting their digestive proteases to the type of host plant ingested and also to the presence of specific defense compounds induced by the natural plant priming molecule Hx and demonstrate that Hx plant priming enhanced Bt insecticidal activity against CPB. We proposed that Hx host-mediated effects on *B. thuringiensis* efficacy might be biotechnologically exploited to engineer durable, broad spectrum plant protection improving CPB pest management and delaying development of insect resistance. Further research would be needed to assess whether the combination of two innocuous and environmentally friendly strategies, such as Bt and Hx, might be cost-effective and/or complement other insect pest control methods.

## Figures and Tables

**Table 1:** Feeding performance of CPB larvae on three solanaceous plants and mortality caused by a 4.3 pmol Cry3Aa toxin dose. Data are the mean values ± SE of three replicates (*n* = 3) of each sixteen larvae.

CPB plant diet	Weight (mg/individual)		% relative weight increase *	Cry3Aa mortality (%)

	Day 0	Day 7		
Potato	50.3 ± 0.8 a	152.4 ± 5.8	203	61 ± 5
Eggplant	51.6 ± 2.1 a	114.1 ± 0.2 b	121	79 ± 8
Tomato	51.2 ± 0.6 a	100.5 ± 4.8 b	96	89 ± 6

* As compared to the initial weight at day 0;

a,b Means followed by the same letter are not significantly different (Student’s *t*-test, *p* > 0.05).

**Table 2:** Mass Spectrometry analysis of CPB BBMV proteins excised from the Coomassie stained 2D-gel of Figure 2B that gave a positive match.

No.	NCBI accession No.	Name	Species	Mascot score	MW (kDa)	No. identified peptides
1	XP_974860	Aconitase	*Tribolium castaneum*	140	86.23	29
2	XP_001663037	Aconitase	*Aedes aegypti*	154	86.41	29
3	XP_974860	Aconitase	*T. castaneum*	120	86.23	29
4	XP_317642	Aconitase	*Anopheles gambiae*	134	85.92	27
5	XP_317642	Aconitase	*A. gambiae*	162	85.92	26
6	XP_624353	Aldo-keto reductase	*Apis mellifera*	124	36.46	5
7	XP_974606	Prohibitin-1	*T. castaneum*	280	30.32	11
8	ABM55486	Intestain D-6	*Leptinotarsa decemlineata*	86	36.75	12
	ABM55485	Intestain D-5	*L. decemlineata*	86	36.76	12
9	AAN77410	Intestain B-2	*L. decemlineata*	76	22.33	6
	AAS20591	Intestain B-11	*L. decemlineata*	73	36.67	6
	AAS20592	Intestain B-12	*L. decemlineata*	73	36.65	6
10	ABM55486	Intestain D-6	*L. decemlineata*	206	36.75	20
	ABM55485	Intestain D-5	*L. decemlineata*	206	36.75	20
	ABM55484	Intestain D-4	*L. decemlineata*	145	36.80	18
	ABM55491	Intestain D-11	*L. decemlineata*	126	10.88	9
	ABM55488	Intestain D-8	*L. decemlineata*	124	10.34	9
	ABM55495	Intestain D-15	*L. decemlineata*	87	29.74	13
